# Opportunities for prevention: a data-linkage study to inform a public health response to youth offending in the Northern Territory, Australia

**DOI:** 10.1186/s12889-021-11645-4

**Published:** 2021-08-30

**Authors:** Vincent Yaofeng He, Bernard Leckning, Catia Malvaso, Tamika Williams, Leanne Liddle, Steven Guthridge

**Affiliations:** 1 Menzies School of Health Research, Building Red 9, Charles Darwin University, Casuarina campus, Ellengowan Drive, Casuarina NT, Northern Territory 0810 Australia; 2 University of Adelaide, Adelaide, South Australia Australia; 3 Aboriginal Justice Unit, Department of the Attorney-General and Justice, Northern Territory Government, Darwin, Northern Territory Australia

**Keywords:** Aboriginal children, Child abuse and neglect, Child maltreatment, Child protection, Crossover children, Data-linkage, Early developmental crime prevention, Youth justice, Youth offending

## Abstract

**Background:**

Numerous studies have demonstrated a strong link between child maltreatment and subsequent youth offending, leading to calls for early intervention initiatives. However, there have been few whole-population studies into the dimensions of statutory child maltreatment responses that can inform these programs. The aim of this study was to investigate the sex-specific association between level and timing of child protection system (CPS) contact and youth offending.

**Methods:**

This retrospective cohort study used linked individual-level records from multiple agencies, for 10,438Aboriginal children born in the Northern Territory between 1999 and 2006. The outcome measure was the first alleged offence. Key explanatory variables were level (no contact through to out-of-home care) and timing (0–4 years, 5–9 years, or both) of CPS contact. The Kaplan–Meier method was used to estimate cumulative incidence and a flexible parametric survival model to estimate hazard ratios (HR).

**Results:**

Children with no record of CPS contact before age 10 had the lowest cumulative incidence of first alleged offence by age 18 (boys: 23.4% [95%CI:21.0–26.1]; girls: 6.6% [95%CI:5.3–8.2]) and those with a record of out-of-home care the highest CI (boys: 45.5% [95%CI:37.0–54.9]; girls: 18.6% [95%CI:13.0–26.2]). The association of CPS contact with the relative risk of a first alleged offence was greatest for children aged 10–13 years and decreased with age. Timing of CPS contact was also associated with increasing cumulative incidence. The relative risk for first alleged offence was generally higher for children with CPS contact, of any type, during both developmental phases including notifications during both phases (boys, HR at age 11: 8.9 [95%CI:4.2–17.2]; girls, HR at age 11: 13.7 [95%CI:3.8–48.9]) and substantiations during both phases (boys, HR at age 11: 17.0 [95%CI:9.6–30.0]; girls, HR at age 11: 54.1 [95%CI:18.1–162]).

**Conclusion:**

The increased risk of offending associated with level and timing of early CPS contact highlights opportunities for a differentiated public health response to improve life trajectories for children and to reduce youth crime. Although children with unsubstantiated notifications of maltreatment do not meet the criteria for a statutory CPS response, the higher risk of offending among these children supports their inclusion in targeted preventive interventions.

**Supplementary Information:**

The online version contains supplementary material available at 10.1186/s12889-021-11645-4.

## Background

Over the past two decades, there has been a growing call for responses to young people who offend which focus on primary prevention and early support in childhood rather than crisis management and intervention after offending has occurred [1–4]. Calls for reform have been supported by a body of research that has demonstrated a greater risk of youth offending among children who have suffered chronic or recurrent child maltreatment [5–7]. However, while these studies provide valuable insights into the child maltreatment experience of children and their interaction with the youth justice system, the focus on children reported to the child protection system (CPS) for child maltreatment is insufficient to inform a comprehensive whole-of-population perspective on the risk of offending for children with varying levels of CPS contact. A related gap in knowledge is that most studies have concentrated on substantiated reports of child maltreatment, despite concerns about the reliability and validity of the substantiation process and arguments for the greater relevance of unsubstantiated notifications when developing prevention strategies [8]. With notifications for maltreatment increasing and CPSs unable to investigate all reports [9], there is a need to establish whether any child protection contact, including unsubstantiated notifications, is associated with an increased risk of offending. There is also a need to confirm reports that there may be different mechanisms and trajectories leading to youth offending, between males and females, and between different ethnic groups [10, 11]. Large-scale population studies are needed to understand the characteristics of CPS contact that can inform interventions for groups of young people on different risk pathways.

A deeper understanding of the association between CPS contact and youth offending is particularly relevant in the Northern Territory (NT) where rates of both are the highest in Australia. In the NT, Aboriginal and Torres Strait Islander children (*hereafter referred* *to respectfully as* *Aboriginal children in accordance with the preference of Aboriginal people in the NT*) comprise 44% of all children [12] yet, in 2018–2019 made up 87% of children in out-of-home care (OOHC) [13] and 96% of all youth justice detainees [12]. In 2017, the *Royal Commission into the Protection and Detention of Children in the Northern Territory* (Royal Commission) reported that the majority of Aboriginal children who had been found guilty of an offence (75%) had prior contact with CPS, which “suggests a trajectory where engagement with child protection services is a foreseeable pathway to later engagement with the youth justice system” [14]. Early contact of NT children with CPS has been previously recognised as an opportunity for prevention beyond the statutory obligation to determine and respond to maltreatment. A public inquiry, in 2010, raised concerns about the CPS adopting a “forensic approach that focuses more on the technicalities of whether harm occurred than on meeting the actual needs of families and children.” The Inquiry report (*Growing Them Strong, Together*, 2010) outlined a public health approach that focused on early intervention, with referral pathways for families to access the necessary services and support including for children with no notifications or unsubstantiated notifications [15]. In framing a response to the over-representation of Aboriginal children in the CPS and youth justice systems, the impact of colonisation and policies of European settlement that has eroded cultural traditions and self-determination cannot be underestimated [16, 17]. The landmark *Bringing Them Home (1997)* report (i.e. *National Inquiry into the Separation of Aboriginal and Torres Strait Islander Children from their Families* [18]) highlighted the intergenerational impacts caused by historical policies of forced assimilation and removal of children that today “make a parent more susceptible to difficulties in raising their own children and increase the likelihood of further intervention by welfare and juvenile justice departments” [19]. While this socio-historical context points to the greater need for Aboriginal children to receive culturally appropriate interventions and support, very little of the evidence generated in Australia has focused on this vulnerable group.

To address the gaps in evidence, especially for Aboriginal children, a whole-of-population data linkage study was designed to investigate the pathways of children through the CPS associated with a higher risk of youth justice involvement. The practical question of whether to include unsubstantiated notifications in the study of the maltreatment-offending link is even more pertinent in jurisdictions, such as the NT, where a high proportion of children have first CPS contact at an early age and have persistent contacts (i.e. 53% of NT Aboriginal children, born in 2009–2010, had CPS contact before age 5 years [20]). Specifically, this study aims to investigate amongst Aboriginal children (a) the association between the level of CPS contact, before age 10 years, with youth offending; and (b) the association between the timing of unsubstantiated and substantiated child protection notifications with youth offending. The findings of this study have the potential to inform a number of relevant policy reforms including the recent Australian *National Agreement on Closing the Gap* [21] and the *NT Government’s Reform Implementation Plan* [22, 23] that respond to the recommendations of the Royal Commission.

## Methods

### Study design and study population

This is a retrospective population-based cohort study that used de-identified, individual-level information linked across multiple administrative datasets. The study design was informed by a systematic review of the methodological features of past studies relating to the maltreatment-offending association [24]. The study population comprised all Aboriginal children born in the NT between 1st January 1999 and 31st December 2006 with at least one record in any of the linked datasets at or after age 10 years. Of the 11,835 children, born during the selection period, 10,438 children were retained in the study after excluding those with no records in administrative datasets at or after age 10 years (1360 children), in OOHC for social support and with no child protection notifications before age 10 years (17 children) or, who died during the study period (20 children).

### Data sources

Data for this study were obtained from an extensive repository of linked administrative datasets established by the Child and Youth Development Research Partnership [25], a collaboration between the Menzies School of Health Research and NT Government agencies. The datasets contain de-identified unit-level information for NT children with individual linkage keys prepared by SA NT DataLink using probabilistic linkage with clerical review for uncertain matches [26]. Three key datasets were used in the study. The NT Perinatal Data Register is a statutory collection of information for all births in the NT. The second key dataset was the statutory record of contacts of children with the CPS. This includes information on notifications (reports) of possible maltreatment, substantiated cases of maltreatment and OOHC placement. The third dataset was the NT Integrated Justice Information System (IJIS) which contains records for individuals charged with an offence including court appearances [27]. Other linked datasets including health and education records were used as a record of continuing NT residence for the study population.

### Study variables

#### Outcome

In all Australian states and territories, the minimum age of criminal responsibility is 10 years, and the upper age limit for legal proceedings within the youth justice system is 17 years, at the time of the offence [28]. The police are usually the first point of contact for young people involved with the youth justice system [29]. After investigation, police can commence a number of legal actions and cautions, including either court actions (with charges to be tried in court) or non-court actions (such as cautions, diversions and infringement notices) [28]. In our study, the outcome of interest was the first alleged offence (hereafter referred to as first offence) committed between 10 and 17 years that resulted in being charged with an offence by police.

#### Explanatory variables: level and timing of CPS contact

In the NT, all reports of suspected child maltreatment are made to the CPS through a central intake team and are recorded as “notifications”. After assessment, notifications which are deemed consistent with maltreatment are referred for investigation. After investigation, notifications are substantiated (hereafter, “substantiations”) where evidence of abuse or neglect has been identified and the child is deemed to be in need of protection [30]. Out-of-home care (OOHC) refers to children placed in overnight care, including with relatives (other than parents). To examine the differential risk of the timing of maltreatment, timing was defined as CPS contact occurring in either early childhood, from 0 to 4 years, or middle childhood, from 5 to 9 years.

Based on this understanding of CPS contact, to meet the first aim of this study the exposure refers to ‘level of CPS contact before age 10’ and was defined as four mutually exclusive categories:

1. ‘No contact’ with CPS before age 10,
2. ‘Notifications Only’ (one or more notifications but no substantiations before age 10),
3. ‘Substantiations Only’ (one or more substantiations but no OOHC placement before age 10), and
4. ‘Out-of-home care’ (at least one substantiation leading to OOHC placement before age 10).

To meet the second aim of the study, we derived the ‘timing of unsubstantiated and substantiated notifications before age 10’ as a categorical variable containing nine mutually exclusive categories based on the combination of timing and level of CPS contact:

1. no record of CPS contact before age 10 (**),
2. one or more unsubstantiated notifications but no substantiations, at age 0–4 only (N*),
3. one or more substantiated notifications, at age 0–4 only (S*),
4. one or more unsubstantiated notifications but no substantiations, at age 5–9 only (*N),
5. one or more substantiated notifications, at age 5–9 only (*S),
6. one or more unsubstantiated notifications but no substantiations, at both age 0–4 and age 5–9 (NN),
7. one or more substantiated notifications at age 0–4 and unsubstantiated notifications at age 5–9 (SN),
8. one or more unsubstantiated notifications at age 0–4 and substantiated notifications at age 5–9 (NS), and
9. one or more substantiated notifications at both age 0–4 and age 5–9 (SS).

#### Potential confounding variables

From the perinatal dataset, we reviewed maternal and birth-related characteristics which have been demonstrated in previous studies to be associated with an increased risk of contact with the youth justice system [31, 32]. The available pregnancy and labour complications data had been previously audited for completeness [33], and on this basis the following were included in the explanatory model: mothers’ Aboriginal status, pregnancy complications due to gestational diabetes, pre-existing diabetes and pre-eclampsia; and, labour complications due to cord prolapse, fetal distress, manual removal of placenta, meconium stained liquor, obstructed labour, postpartum haemorrhage and “other obstetric complications”.

### Statistical analysis

In our study, the analyses were stratified by sex based on literature suggesting different mechanisms and developmental trajectories leading to offending between males and females [10, 11]. Survival analysis was used to investigate the association between contact with the CPS and a first offence. Survival time was defined as the time in years from the tenth birthday to the occurrence of first offence, children who did not have a record of first offence were censored at 31 December 2017 or 18 years of age. The time-scale for the survival analysis was the age of children in years (as continuous variable).

The risk of first offence by groups of children with different levels of exposure was estimated as both absolute and relative risk [34–36], with absolute risks reported as cumulative incidence and relative risks as hazard ratios. The cumulative incidence is the cumulative proportion of new cases of first offence reported from 10 years of age onward; and, hazard ratios are the ratio of hazard rates for children with CPS contact compared to those children with no CPS contact, before age 10 years.

To calculate the cumulative incidence, the Kaplan–Meier estimator method was used. In this study, the Kaplan-Meier failure function is equivalent to the cumulative incidence estimated using a competing risk model (that considers death as a competing risk), as we excluded the 20 children who died in the study period. To estimate the hazard ratios, a flexible parametric survival model was used, in which the functional form/shape of the baseline hazard distribution was modelled as a restricted cubic spline [37, 38]. The proportional hazards assumption was tested using the scaled Schoenfeld residuals against time. Where model covariates violated the proportional hazards assumption, time-varying effects were modelled using spline functions. The Akaike Information Criterion (AIC) and Bayesian Information Criterion (BIC) were used to determine the optimum number of spline knots of the baseline hazard and the optimum number of the knots of time-varying effects. In the multivariable regression, we adjusted for confounding influences. The Royston R^2^ measure was used to explain the proportion of variation in the outcome explained by the model [39]. All analyses were conducted using Stata for Windows, Version 15 [40].

### Ethical approval

The study was approved by the Human Research Ethic Committee of the NT Department of Health and the Menzies School of Health Research (HREC-2016-2708) and was supported by the Child Health Division Indigenous Reference Group which includes independent Aboriginal community members.

## Results

### Characteristics of study cohort

The characteristics of all 10,438 children in the study cohort are presented by sex in Table 1 (with characteristics of children with first offence presented in Additional file 1: Table S1). About half of all the children had no record of CPS contact before age 10 years (boys: 53.6%; girls: 54.1%). About a quarter of the study cohort had a record of CPS contact for an unsubstantiated notification only (boys 24.5%, girls 24.1%) and smaller proportions had a record of CPS contact for either substantiated notifications (boys:14.7%, girls: 14.0%) or out-of-home care (boys:7.2%, girls:7.8%).

**Table 1:** Characteristics of study cohort (Aboriginal children born between 1999 and 2006 and in NT at age 10 years)

	Boys n (%)	Girls n (%)
Total	5403(100%)	5035(100%)
Level of CPS contact		
No contact	2894(53.6%)	2722(54.1%)
Notification only	1324(24.5%)	1215(24.1%)
Substantiation only	794(14.7%)	703(14.0%)
Out-of-home care	391(7.2%)	395(7.8%)
Timing and level of CPS contact		
N* (one or more unsubstantiated notifications but no substantiations at age 0–4, only)	252(4.7%)	237(4.7%)
S* (one or more substantiated notifications at age 0–4, only)	187(3.5%)	189(3.8%)
*N (one or more unsubstantiated notifications but no substantiations at age 5–9, only)	830(15.4%)	768(15.3%)
*S (one or more substantiated notifications at age 5–9, only)	361(6.7%)	315(6.3%)
NN (one or more unsubstantiated notifications but no substantiations, at both age 0–4 and age 5–9)	272(5.0%)	247(4.9%)
SN (one or more substantiated notifications at age 0–4 and unsubstantiated notifications at age 5–9)	203(3.8%)	211(4.2%)
NS (one or more unsubstantiated notifications at age 0–4 and substantiated notifications at age 5–9)	198(3.7%)	159(3.2%)
SS (one or more substantiated notifications at both age 0–4 and age 5–9)	206(3.8%)	187(3.7%)
Indigenous status of mother		
Non-Aboriginal	589(10.9%)	515(10.2%)
Aboriginal	4814(89.1%)	4520(89.8%)
Health district of mother’s residence prior to birth		
Darwin Rural	1046(19.4%)	932(18.5%)
Darwin Urban	961(17.8%)	920(18.3%)
Katherine	965(17.9%)	907(18.0%)
East Arnhem	901(16.7%)	819(16.3%)
Barkly	354(6.6%)	311(6.2%)
Alice Springs Urban	410(7.6%)	390(7.7%)
Alice Springs Rural	766(14.2%)	756(15.0%)
Maternal age at birth		
> =35	302(5.6%)	299(5.9%)
< 20	1537(28.4%)	1478(29.4%)
20–24	1679(31.1%)	1487(29.5%)
25–29	1188(22.0%)	1134(22.5%)
30–34	697(12.9%)	637(12.7%)
Parity		
0	1714(31.7%)	1664(33.0%)
1–2	2360(43.7%)	2096(41.6%)
> =3	1327(24.6%)	1272(25.3%)
Low birth weight (< 2500 g)		
No	4769(88.3%)	4355(86.5%)
Yes	634(11.7%)	680(13.5%)
Gestation (in weeks)		
> =37wk	4665(86.3%)	4353(86.5%)
< 32wk	139(2.6%)	141(2.8%)
33-36wk	599(11.1%)	541(10.7%)
Complication in pregnancy		
No	4865(90.0%)	4531(90.0%)
Yes	538(10.0%)	504(10.0%)
Complication in labour		
No	3204(59.3%)	3063(60.8%)
Yes	2199(40.7%)	1972(39.2%)
Other obstetric complication		
No	4537(84.0%)	4211(83.6%)
Yes	866(16.0%)	824(16.4%)

### Level of contact with the child protection service by age 10

#### Cumulative incidence

**Fig. 1:**
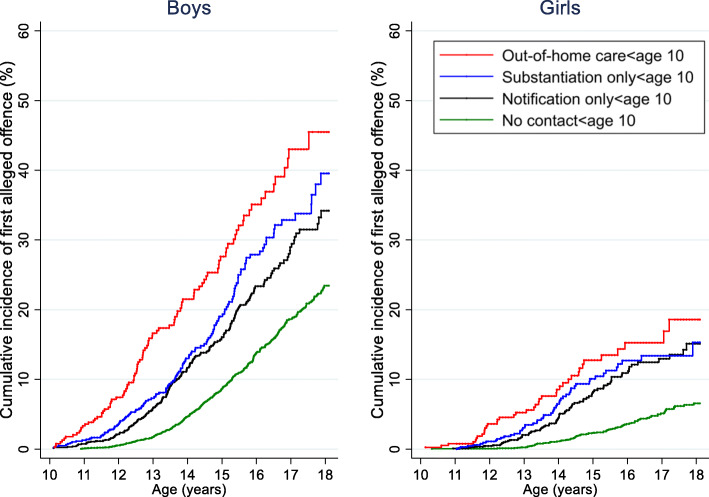
Cumulative incidence of first alleged offence by level of CPS contact

For both Aboriginal boys and girls, there was a gradient of increasing risk for a first offence as level of CPS contact (before age 10 years) increased (Fig. 1 with full results presented in Additional file 1: Table S2). For boys, the lowest cumulative incidence of first offence by 18 years of age was among those with no record of CPS contact (23.4, 95% confidence interval (CI): 21.0–26.1) and highest was for those with a record of OOHC (45.5, 95%CI: 37.0–54.9). Among girls, the group with the lowest cumulative incidence of first offence by 18 years of age was the group with no record of CPS contact (6.6, 95%CI: 5.3–8.2) and highest was the group with record of OOHC (18.6, 95%CI:13.0–26.2).

#### Hazard ratio

**Fig. 2:**
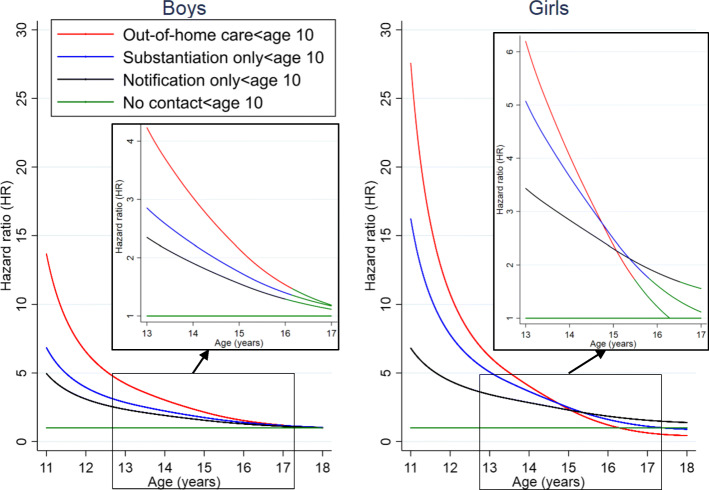
Relative risk over time of first alleged offence by level of CPS contact. Notes: 1. Adjusted for birth-cohort effects, maternal and birth characteristics and residential location of mother at time of birth. 2. or subgraphs, the lines become green when the 95% confidence intervals include the null value of 1, in which the reference population is the children with no record of CPS contact

Figure 2 presents the hazard ratios for boys and girls, by level of CPS contact, estimated by multivariable regression after controlling for confounding (full results in Additional file 1: Table S4). The analysis demonstrates the association between level of CPS contact (before age 10) and first offence varies with age, with the greatest relative risk observed for boys and girls with a first offence between 10 and 14 years after which the estimates of relative risk decrease with age. By age 11 years, the hazard ratios for Aboriginal boys with notifications only, substantiations or OOHC were 5.0 (95%CI: 3.1–8.0), 6.9 (95%CI: 4.2–11.2) and 13.7 (95%CI: 8.3–22.5) respectively compared with boys with no record of CPS contact before age 10 years. By age 14 years, the corresponding hazard ratios for boys reduced to 1.9 (95%CI: 1.6–2.3), 2.2 (95%CI: 1.8–2.8) and 3.0 (95%CI: 2.4–3.9) respectively. Amongst girls, the estimates of relative risk of first offence associated with CPS contact (before age 10 years) are higher compared to boys. By age 11 years, the hazard ratios for those Aboriginal girls with a record of notifications only, substantiations or OOHC before age 10 years, were 6.8 (95%CI: 2.6–18.2), 16.3 (95%CI: 6.0–44.0) and 27.6 (95%CI: 10.5–72.8). By age 14 years, the corresponding hazard ratios for girls were 2.8 (95%CI: 2.0–4.0), 3.7 (95%CI: 2.5–5.3) and 4.0 (95%CI: 2.6–6.2) respectively. By late adolescence, around age 17 years, there is no evidence for difference in risk of first offence among those remaining boys and girls with a record of CPS contact compared to the remaining boys and girls with no record of CPS contact before age 10 (Fig. 2).

The Royston R^2^ for the multivariable model was 0.59 for the boys and 0.67 for the girls (Additional file 1: Table S5), which confirms that the model explains a high proportion of variation in the outcome.

### Association between the timing and level of CPS contact, before age 10 years, and first offence

#### Cumulative incidence

**Fig. 3:**
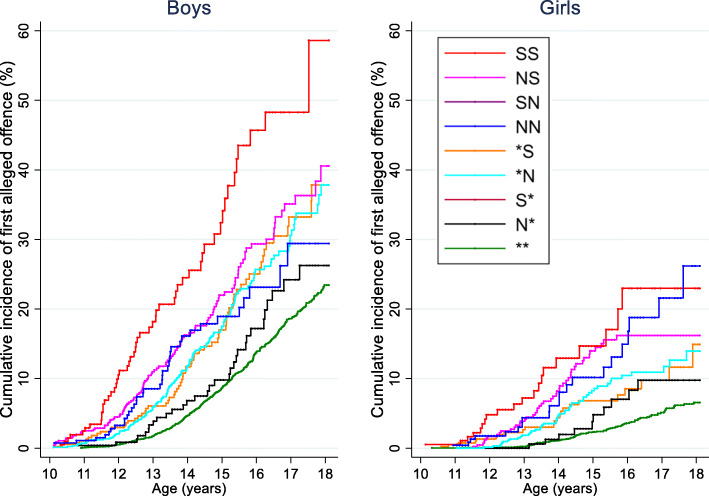
Cumulative incidence of first alleged offence by level and timing of CPS contact. Note: ** (no record of CPS contact before age 10); N* (one or more unsubstantiated notifications but no substantiations at age 0–4, only); S* (one or more substantiated notifications at age 0–4, only); *N (one or more unsubstantiated notifications but no substantiations at age 5–9, only); *S (one or more substantiated notifications at age 5–9, only); **NN** (one or more unsubstantiated notifications but no substantiations, at both age 0–4 and age 5–9); **SN** (one or more substantiated notifications at age 0–4 and unsubstantiated notifications at age 5–9); **NS** (one or more unsubstantiated notifications at age 0–4 and substantiated notifications at age 5–9); **SS** (one or more substantiated notifications at both age 0–4 and age 5–9)

Figure 3 presents the cumulative incidence of first offence for boys and girls based on timing of CPS contact in early (0–4 years) or middle childhood (5–9 years) and level of CPS contact before age 10 (full results in Additional file 1: Table S2). For boys, the highest cumulative incidence of first offence by 18 years of age was among those with a record of substantiations in both age groups (**SS** group, 58.6%; 95%CI: 39.6–78.7) and those with notifications only at 0 to 4 years and substantiated notifications at age 5 to 9 years (**NS** group, 46.0%; 95%CI: 33.2–61.0). The lowest cumulative incidence was among boys with no record of CPS contact (** group, 23.4%; 95%CI:21.0–26.1). The higher cumulative incidence of first offence was also evident for girls with CPS contact at both ages although the order of the exposures was different to boys. The highest cumulative incidence of first offence, up to age 18 years, was for girls with a record of notification at age 0 to 4 years and substantiated notifications at age 5 to 9 years (**NS** group, 30.9%; 95%CI:21.1–43.9), followed by those with notifications only in both stages of childhood (**NN** group, 26.2%; 95%CI:15.7–41.7) and substantiations at both stages of childhood (**SS** group, 23.0%: 95%CI 14.0–36.3). The group with the lowest cumulative incidence was girls with no record of CPS contact (** group, 6.6%; 95%CI:5.3–8.2). Whilst there was some variation in the overall pattern of level and timing of CPS contact between Aboriginal boys and girls, it should be noted that for both groups, we found higher estimates of absolute risk of a first offence among those children with CPS contact in both stages of childhood than those with CPS contact in only one stage of childhood.

#### Hazard ratio

**Fig. 4:**
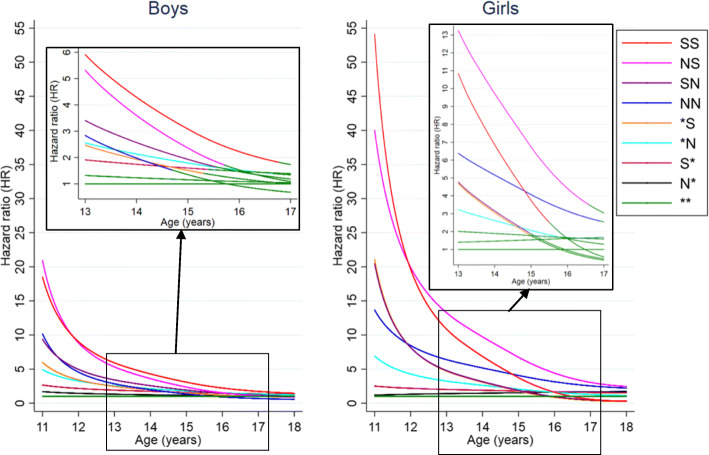
Relative risk over time of first alleged offence by level and timing of CPS contact. Notes: 1. Adjusted for birth-cohort effects, maternal and birth characteristics and residential location of mother at time of birth. 2. * (no record of CPS contact before age 10); N* (one or more unsubstantiated notifications but no substantiations at age 0–4, only); S* (one or more substantiated notifications at age 0–4, only); *N (one or more unsubstantiated notifications but no substantiations at age 5–9, only); *S (one or more substantiated notifications at age 5–9, only); **NN** (one or more unsubstantiated notifications but no substantiations, at both age 0–4 and age 5–9); **SN** (one or more substantiated notifications at age 0–4 and unsubstantiated notifications at age 5–9); **NS** (one or more unsubstantiated notifications at age 0–4 and substantiated notifications at age 5–9); **SS** (one or more substantiated notifications at both age 0–4 and age 5–9). 3. For subgraphs, the lines involvement become green when the 95% confidence intervals include the null value of 1, in which the reference population is the children with no record of CPS contact before age 10

Figure 4 presents the hazard ratios over time illustrating the relative risk of first offence by timing and level of CPS contact (full results in Additional file 1: Table S4). Similar to the analysis in the previous sub-section, the multivariable regression suggested that the greatest hazard ratio for CPS contact was for boys and girls aged 10 to 14 years at first offence and that the effect is greater for girls than boys.

All groups of boys with CPS contact before age 10, except boys who had only unsubstantiated notifications in early childhood (N* group), had evidence of higher relative risk of a first offence at ages 10 to 14 years, compared with boys with no record of CPS contact (Additional file 1: Table S4). The greatest relative risk of first offence is observed at age 11 years for boys with a history of notifications in early childhood and substantiations in middle childhood (HR 20.9; 95% CI: 12.0–36.5). Boys with a history of substantiations in both early and middle childhood also had an elevated risk of a first offence by age 11. However, as can be seen by the declining slopes of the hazard ratio plots in Fig. 4, the relative risk of CPS contact for all patterns of involvement are greatly moderated over time such that there is no evidence for a difference in risk of first offence, among remaining boys, by 18 years of age.

For Aboriginal girls, the general patterns of increased hazard ratios are similar to boys, but relative risks are substantially greater. The estimate of the greatest relative risk was found amongst Aboriginal girls with a history of substantiations in both early and middle childhood, who were over 50 times more likely (HR 54.1; 95% CI: 18.1–162.0) of a first offence by age 11 compared to those with no record of CPS contact. This was followed by girls with notifications in early childhood and substantiations in middle childhood who were 40 times more likely to have a first offence by age 11 (HR 40.0; 95% CI: 11.5–140.0) compared to their peers with no CPS contact. For girls, as was observed for boys, the differences in the relative risk of first offence associated with CPS contact declined with increasing age.

It should also be noted that across all analyses, children with any record of CPS contact, irrespective of the level and/or timing, were associated with a higher risk of first offence than children with no record of CPS contact. Importantly, this increased risk includes notifications that were not investigated, which are associated with between 2 and 14 times greater risk of a first offence.

The Royston R^2^ for the multivariable model was 0.58 for the boys and 0.69 for the girls (Additional file 1: Table S5), which again indicates that the model explains a substantial proportion of the variation in the outcome of first offence in both boys and girls.

## Discussion

### Summary of key findings

This is the first known population-based study of contact by NT Aboriginal children with both the child protection and youth justice systems. The results reinforce findings from other studies that child maltreatment is a major risk factor for youth justice system contact. The results also address a gap in research by demonstrating no substantial difference in the risk of first offence for children with unsubstantiated notifications compared to children with substantiated notifications. Instead, any record of CPS contact in both early and middle childhood was found to be strongly associated with a higher risk of youth offending, suggesting that cumulative effects of persistent family adversity and/or maltreatment experiences are important to understanding youth offending behaviour. Our study also reports a greater relative risk of CPS contact on youth justice system contact for girls than boys. While girls have a lower absolute risk of offending than boys, the relative risk of first offence associated with CPS contact is greater for the girls than boys. This is generally consistent with research in this area that shows that boys are far more likely to have contact with the justice system [12]. However, our results suggest that, relative to boys, girls with CPS contact are at increased risk for justice system contact., suggesting that girls are more affected by maltreatment experiences in childhood. These findings highlight the importance of comprehensive gender-responsive early support and intervention programmes that address the multiple and complex needs of vulnerable children involved with both child protection and youth justice services.

### Children in out-of-home care (OOHC)

Our study found that children with a history of OOHC had the highest risk of first offence amongst the four levels of CPS contact. After controlling for a range of potential confounders, children who had experienced placement in OOHC before age 10 had a higher risk of first offence from age 10–15 years. This may be partly explained by greater prevalence of behavioural and psychological problems observed amongst children in OOHC [41], or relatedly by the severity of maltreatment that led to their placement, which may also be compounded by negative placement experiences. There is evidence from other studies to suggest that placement instability (frequent placement changes) [42–45] and some forms of OOHC placement, including residential care and group homes can also increase the risk of offending behaviour [43, 44, 46, 47]. There is also research that suggests that the behaviour of young people in OOHC is more likely to come to the attention of the police [48, 49]. Together, these findings highlight the urgent need to ensure that OOHC services are responsive to the complex needs of these children and that preventive practices are in place to ensure that trauma-related behaviours are not unnecessarily criminalised.

### Similar risk of children with substantiated and unsubstantiated notification

Our results demonstrate that the risk of a first offence for children with a record of one or more unsubstantiated notifications is more likely to be similar to children with substantiated notifications than to children with no CPS contact, before age 10 years. As has been highlighted in similar studies [50–52], while the threshold for substantiation may conform to a threshold for a statutory child protection response it is not sufficiently sensitive to differentiate risk for youth offending in the population. Drake’s ‘harm/evidence model’ provides a two-dimensional theoretical framework that may help to clarify these findings [53]. Under such a model, there might be unsubstantiated notifications that indicate high levels of harm but for which there is insufficient evidence, moderate levels of harm that fail to meet the threshold of the agency guidelines or legislation, even unsubstantiated notifications that could be “serious warning signs of future problems that are not, in themselves, technically maltreatment” [50]. The inclusion of unsubstantiated notifications in this study has provided clearer insight into this under-recognised pathway into youth offending. This finding is also important when planning preventive interventions for which a record of CPS contact, at any level, is an important marker for inclusion in targeted programs.

### Persistence and timing of CPS contact

Although our findings point to the importance of including unsubstantiated notifications in studies of contact with the youth justice system, other dimensions of CPS contact such as persistence and timing of notifications and substantiations appear to be equally important. The findings of our study suggest that the persistence and timing of maltreatment have greater effects on subsequent contact with the youth justice system than whether or not a notification is substantiated. For both boys and girls, the groups of children with the highest risk of first offence were those children who had notifications in early childhood and substantiations in middle childhood (the **NS group**) or substantiated notifications in both periods of childhood (the **SS group**). Of note, is that after age 14 years, those girls with unsubstantiated notification in both periods of childhood (the **NN group**) had a relatively higher risk of first offence than those children with no record of CPS contact before age 10 years.

Previous studies have found that adolescent-limited maltreatment, that is maltreatment that occurs exclusively in adolescence (as often defined by substantiations during this period), is a stronger risk factor for youth offending behaviour than maltreatment limited to the childhood years. However, our findings highlight the potential risk of misclassification when unsubstantiated notifications are not included in the analysis of the link between child maltreatment and youth offending. For example a child with a record of an unsubstantiated notification in early childhood and substantiated notification in middle childhood may be incorrectly classified in a ‘middle childhood limited maltreatment’ group, while children that have substantiations at early childhood and unsubstantiated notifications at middle childhood, could be classified in a ‘early childhood limited maltreatment’ group; when in reality, both groups of children may be more accurately considered to have experienced ‘persistent maltreatment’, a group that has higher risk of adverse outcomes and greater need for support. In a population with a high prevalence of youth offending, it is important to identify the different trajectories of CPS involvement in childhood that can better inform the timing and appropriateness of statutory responses and preventive interventions that contribute to reducing the risk of youth offending. In addition, the majority of young people who have contact with CPS before age 10 never have contact with the justice system, and further research should examine the protective and resilience factors that prevent or mitigate the associations between early contact with the CPS and justice system.

### Implications

Our study has several important implications. First, the high level of CPS contact of NT Aboriginal children, who are later at risk for contact with the youth justice system, presents an opportunity for early developmental crime prevention [54]. Prevention initiatives should not only be targeted towards families with children who have substantiated maltreatment but should be expanded to include those with unsubstantiated notifications. Children with unsubstantiated notifications are also at increased risk of contact with the youth justice system, which indicates the need for family support services for these children even in the absence of meeting the threshold for a statutory child protection response [9]. Although there is scant evaluation [55] of either general or Aboriginal-specific prevention programs in Australia [4, 56, 57], internationally there has been strong evidence for family-based programs (such as behavioural parent training) in reducing delinquency and antisocial behavior in children [58] and for family-focused interventions that adopt a population level approach to parenting support [59]. In Canada, the National Crime Prevention Centre has built a resource containing ‘promising’ programs for Aboriginal populations [60, 61]. One example is the Kwanlin Dun First Nation’s Project, a family-focused prevention program that targets parents with children between the ages of 0–6 years who are considered at high risk of crime and victimization with the aim of reducing the risk factors associated with crime by incorporating culturally specific components, including recognition of the extended family and various cultural preferences, into family home visitations [62]. An evaluation of this program found that only 5% of the program clients reported contact with the CPS during the time of study, compared with 35% of the comparison group [62]. Considering the high social and financial costs of criminal justice in the NT, there is a great need for effective crime prevention programs [54] through early intervention programs that prevent CPS contact.

The second implication is the demonstration of the utility of linked administrative data to identify groups of children who may benefit from early support and intervention. Linked administrative data can also be used to develop a tool, using a risk algorithm, that can be applied to identify groups of children who may benefit from scaled support to reduce contact in the criminal justice system. Such a tool can take account of cultural, gender and ethnic differences that are associated with the over-representation of Aboriginal children in both systems [63].

The third implication is the importance of interagency collaboration, corroborating the recommendations of the Royal Commission which also stressed the need to develop a workforce equipped to deal with the complex needs of children who crossover between the child protection and youth justice systems [14]. This is particularly important in settings with relatively high prevalence of both child maltreatment reports and youth offending behaviour, such as the NT where it has also been established that community-level influences contribute to the high risk of youth offending [64]. This and other similar studies also reinforce a separate recommendation of the Royal Commission calling for more research into place-based strategies for community safety and crime prevention [63] to inform a whole-of-community approach and inter-agency collaboration to child protection and youth justice.

The fourth implication is the need for broader public health initiatives that recognise the complex range of factors (individual, familial and contextual influences) that underpin both child maltreatment and youth offending. To address these contextual influences as well as the range of adverse health and social outcomes associated with CPS contact, it is important that early intervention initiatives designed to be implemented through the CPS be embedded within a more general public health framework that recognises the multiple and complex needs of vulnerable children. Importantly, it is within a public health framework that the socio-historical context that underpins the over-representation of Aboriginal children in both child protection and youth justice services can be recognised and inform culturally appropriate CPS responses and associated preventive interventions.

### Strengths and limitations of study

There are a number of strengths of our study. First, the use of population-level linked data provides comprehensive coverage and representativeness of the study population. Second, the analysis has focussed on a high need population and was stratified by sex resulting in findings that support culturally relevant and sex-specific responses. The analysis also incorporates an appropriate comparison group of Aboriginal children with no record of CPS contact.

There are also limitations to our study. Firstly, the analysis has been restricted to the outcome of first event and not subsequent events, which limits the reporting to prevalence of first offence and does not include assessment of the frequency of youth offending [32] nor the analysis of the relationship of CPS contact with varying types of offences. Secondly, to maintain the correct temporal order between CPS contact and youth offending required for longitudinal analysis, we have confined our analysis to CPS contact before age 10 years. CPS contact with older children may also be an important opportunity for intervention but was not explored in this study. A third limitation is the outcome measure for youth offending is based on court data and does not use police data which contains additional information on apprehension and youth diversion. A fourth limitation is information on parental factors, which are potential confounders, were not available for inclusion in this study. A final limitation is the generalisability of our results. This study was undertaken with a study population living in a complex setting which includes intergenerational disadvantage and poverty. Care is needed if applying the results to other populations including other First Nation populations.

## Conclusion

Our study confirms that, for both boys and girls, CPS contact is a major risk for subsequent contact with the youth justice system and demonstrates that the risk of first offence likely increases with the level of CPS contact. The risk is further increased when CPS contact occurs during both early and middle childhood. The study also found similar risk of first offence for children with substantiated and unsubstantiated notifications, suggesting the need to include unsubstantiated notifications when responding to the link between CPS contact and youth offending. Our study highlights the opportunity for comprehensive early intervention programmes that attempt to address the multiple individual, familial and contextual factors that contribute to the complex needs of children involved with both child protection and youth justice services.

## Supplementary Information

**Additional file 1.**

## Data Availability

The study datasets contain sensitive personal information and are held on a secure cloud-based server with restricted access. Access requires the approval of the ethics committee and data custodians.

## Abbreviations

CPS: Child protection system
HR: Hazard ratio
IJIS: Integrated Justice Information System
NT: Northern Territory
OOHC: Out-of-home care
**: No record of CPS contact before age 10)
N*: One or more unsubstantiated notifications but no substantiations at age 0–4, only
S*: One or more substantiated notifications at age 0–4, only
*N: One or more unsubstantiated notifications but no substantiations at age 5–9, only)
*S: One or more substantiated notifications at age 5–9, only)
NN: One or more unsubstantiated notifications but no substantiations, at both age 0–4 and age 5–9
SN: One or more substantiated notifications at age 0–4 and unsubstantiated notifications at age 5–9
NS: One or more unsubstantiated notifications at age 0–4 and substantiated notifications at age 5–9
SS: One or more substantiated notifications at both age 0–4 and age 5–9.

## References

[1] Moore MH (1995). Public health and criminal justice approaches to prevention. Crime Justice.

[2] Royal Commission and Board of Inquiry into the Protection and Detention of Children in the Northern Territory, ‘Final Report’, Volume 3B, Chapter 38. 2017. https://www.royalcommission.gov.au/system/files/2020-09/Volume%203B.pdf Accessed 20 Aug 2021.

[3] Royal Commission and Board of Inquiry into the Protection and Detention of Children in the Northern Territory, ‘Final Report’, Volume 2B, Chapter 27. 2017. https://www.royalcommission.gov.au/system/files/2020-09/Volume%202B.pdf Accessed 20 Aug 2021.

[4] Higgins D & Davis K. 2014, Law and justice: prevention and early intervention programs for indigenous youth (resource sheet no. 34), Australian Institute of Health and Welfare & Melbourne: Australian Institute of Family Studies. https://www.aihw.gov.au/getmedia/85dd676d-62ab-47cf-8a01-a1847a05a17a/ctg-rs34.pdf.aspx. Accessed 20 Aug 2021.

[5] Malvaso CG, Delfabbro PH, Day A (2016). Risk factors that influence the maltreatment-offending association: a systematic review of prospective and longitudinal studies. Aggress Violent Behav.

[6] Braga T, Gonçalves LC, Basto-Pereira M, Maia Â (2017). Unraveling the link between maltreatment and juvenile antisocial behavior: a meta-analysis of prospective longitudinal studies. Aggress Violent Behav.

[7] Malvaso CG, Delfabbro PH, Day A (2017). Child maltreatment and criminal convictions in youth: the role of gender, ethnicity and placement experiences in an Australian population. Child Youth Serv Rev.

[8] Hussey JM, Marshall JM, English DJ, Knight ED, Lau AS, Dubowitz H, Kotch JB (2005). Defining maltreatment according to substantiation: distinction without a difference?. Child Abuse Negl.

[9] Royal Commission and Board of Inquiry into the Protection and Detention of Children in the Northern Territory. ‘Final Report’, Volume 3A, Chapter 32. 2017. https://www.royalcommission.gov.au/system/files/2020-09/Volume%203A.pdf Accessed 20 Aug 2021.

[10] Topitzes J, Mersky JP, Reynolds AJ (2011). Child maltreatment and offending behavior: gender-specific effects and pathways. Crim Justice Behav.

[11] Ferrante AM (2013). Assessing gender and ethnic differences in developmental trajectories of offending. Aust N Z J Criminol.

[12] Australian Institute of Health and Welfare (AIHW) (2020). Youth justice in the Northern Territory 2018–19.

[13] Australian Institute of Health and Welfare (AIHW) (2020). Child protection Australia 2018–19. Child welfare series no. 72. Cat. no. CWS 74.

[14] Royal Commission and Board of Inquiry into the Protection and Detention of Children in the Northern Territory. Final Report, Volume 3B, Chapter 35. 2017. https://www.royalcommission.gov.au/system/files/2020-09/Volume%203B.pdf Accessed 20 Aug 2021.

[15] Northern Territory Government. Growing them Strong, Together: Promoting the safety and wellbeing of the Northern Territory’s children, Report of the Board of Inquiry into the Child Protection System in the Northern Territory 2010, M. Bamblett, H. Bath and R. Roseby, Northern Territory Government, Darwin. 2010. https://digitallibrary.health.nt.gov.au/prodjspui/bitstream/10137/459/1/CPS%20Report%202010.pdf Accessed 20 Aug 2021.

[16] Nielsen MO, Robyn L (2003). Colonialism and criminal justice for indigenous peoples in Australia, Canada, New Zealand and the United States of America.

[17] Sinha V, Trocmé N, Fallon B, MacLaurin B (2013). Understanding the investigation-stage overrepresentation of first nations children in the child welfare system: an analysis of the first nations component of the Canadian incidence study of reported child abuse and neglect 2008. Child Abuse Negl.

[18] Wilkie M (1997). Bringing them home: report of the national inquiry into the separation of Aboriginal and Torres Strait islander children from their families: human rights and equal opportunity commission.

[19] Cunneen C, Libesman T (2000). Postcolonial trauma: the contemporary removal of indigenous children and young people from their families in Australia. Aust J Soc Issues.

[20] He V, Guthridge S, Leckning B (2019). From from birth to five: a multiagency data-linkage study to inform a public health response to child protection in the Northern Territory.

[21] Australian Government (2020) (2020). National agreement on closing the gap.

[22] Northern Territory Government. Safe, Thriving and Connected - Generational Change for Children and Families: The Northern Territory Government’s plan to implement reforms for better supporting children, young people and families experiencing vulnerability and delivering the recommendations of the Royal Commission into the Protection and Detention of Children in the Northern Territory 2018–2023. 2018. https://rmo.nt.gov.au/__data/assets/pdf_file/0005/498173/Safe,-Thriving-and-Connected-Implementation-Plan-Web.pdf. Accessed 20 Aug 2021.

[23] Northern Territory Government. Child Protection and Youth Justice Reform-Implementation and Reporting Framework. NT Royal Commission. Exh.469.342. Page 17. 2017. http://content.webarchive.nla.gov.au/gov/wayback/20180615091700/https://childdetentionnt.royalcommission.gov.au/NT-public-hearings/Documents/evidence-2017/evidence2june/Exh-469-342.pdf. Accessed 20 Aug 2021.

[24] Malvaso CG, Delfabbro P, Day A (2015). The maltreatment–offending association: a systematic review of the methodological features of prospective and longitudinal studies. Trauma Violence Abuse.

[25] He V, Guthridge S, Leckning B (2019). Protection and justice: a study of the crossover of NT children in two services.

[26] SA-NT DataLink: Supporting Health (2021). Social and economic research, education and policy in South Australia and the Northern Territory.

[27] Yick J (2016). An analysis of the association between criminal behavior and experience of maltreatment as a child in the Northern Territory.

[28] Australian Institute of Health and Welfare (AIHW) (2019). Youth Justice in Australia 2017–18. Cat. no. JUV 129.

[29] Productivity Commission (2018). Report on government services, chapter 17: youth justice services. Productivity Commission, Canberra.

[30] Australian Institute of Family Studies (AIFS). Child Family Community Australia (CFCA). Child abuse and neglect statistics. Melbourne; 2017. https://aifs.gov.au/cfca/publications/child-abuse-and-neglect-statistics. Accessed 20 Aug 2021

[31] Jones J (2018). Exploring the pathways to contact with juvenile justice in Aboriginal and Torres Strait islander children: developing a profile of the risk and protective factors to support a strategy for change.

[32] Ferrante A (2013). Dimensions of delinquency: exploring group differences in the prevalence and frequency of offending; a linkage based study of offending in the Western Australian Population.

[33] Li L, O’Neil L. Mothers and Babies 2016: Northern Territory Midwives’ Collection. Department of Health, Darwin, 2019. https://digitallibrary.health.nt.gov.au/prodjspui/bitstream/10137/8089/1/Mothers%20and%20Babies%20Report%202016.pdf Accessed 20 Aug 2021.

[34] Noordzij M, van Diepen M, Caskey FC, Jager KJ (2017). Relative risk versus absolute risk: one cannot be interpreted without the other. Nephrol Dial Transplant.

[35] Citrome L (2010). Relative vs. absolute measures of benefit and risk: what’s the difference?. Acta Psychiatr Scand.

[36] Oliver Schmidt C, Kohlmann T (2008). Risk quantification in epidemiologic studies. Int J Public Health.

[37] Lambert PC, Royston P (2009). Further development of flexible parametric models for survival analysis. Stata J.

[38] Royston P, Lambert PC (2011). Flexible parametric survival analysis using Stata: beyond the cox model.

[39] Royston P (2006). Explained variation for survival models. Stata J.

[40] StataCorp. Stata Statistical Software: Release 15. College Station, TX: StataCorp LLC; 2017.

[41] Malvaso CG, Delfabbro PH, Day A, Nobes G (2019). Young people under youth justice supervision with varying child protection histories: an analysis of group differences. Int J Offender Ther Comp Criminol.

[42] DeGue S, Spatz WC (2009). Does out-of-home placement mediate the relationship between child maltreatment and adult criminality?. Child Maltreat.

[43] Goodkind S, Shook JJ, Kim KH, Pohlig RT, Herring DJ (2013). From child welfare to juvenile justice: race, gender, and system experiences. Youth Violence Juvenile Justice.

[44] Ryan J, Hong JS, Herz D, Hernandez PM (2010). Kinship foster care and the risk of juvenile delinquency. Child Youth Serv Rev.

[45] Yampolskaya S, Chuang E (2012). Effects of mental health disorders on the risk of juvenile justice system involvement and recidivism among children placed in out-of-home care. Am J Orthop.

[46] Ryan J (2012). Substitute care in child welfare and the risk of arrest: does the reason for placement matter?. Child Maltreat.

[47] Ryan JP, Marshall JM, Herz D, Hernandez PM (2008). Juvenile delinquency in child welfare: investigating group home effects. Child Youth Serv Rev.

[48] McFarlane K (2018). Care-criminalisation: the involvement of children in out-of-home care in the New South Wales criminal justice system. Aust N Z J Criminol.

[49] Cashmore J (2011). The link between child maltreatment and adolescent offending: systems neglect of adolescents. Fam Matters.

[50] Kohl PL, Jonson-Reid M, Drake B (2009). Time to leave substantiation behind:findings from a national probability study. Child Maltreat.

[51] Jonson-Reid M (2002). Exploring the relationship between child welfare intervention and juvenile corrections involvement. Am J Orthop.

[52] Leiter J, Myers KA, Zingraff MT (1994). Substantiated and unsubstantiated cases of child maltreatment: do their consequences differ?. Soc Work Res.

[53] Drake B (1996). Unraveling “unsubstantiated”. Child Maltreat.

[54] Farrington DP (1994). Early developmental prevention of juvenile delinquency. Crim Behav Ment Health.

[55] Public Safety Canada (2017). Evidence-based crime prevention: scientific basis, trends, results and implications.

[56] Richards K, Rosevear L, Gilbert R (2011). Promising interventions for reducing indigenous juvenile offending.

[57] Stewart J, Hedwards B, Richards K, Willis M, Higgins D (2014). Indigenous youth justice programs evaluation. Special reports.

[58] Farrington DP, Welsh BC (2003). Family-based prevention of offending: a meta-analysis. Aust N Z J Criminol.

[59] Sanders MR (2012). Development, evaluation, and multinational dissemination of the triple P-positive parenting program. Annu Rev Clin Psychol.

[60] Public Safety Canada (2008). Promising and model crime prevention programs.

[61] Public Safety Canada (2011). Promising and model crime prevention programs volume II.

[62] Public Safety Canada (2007). Healthy families project and kwanlin dun first nation’s project: evaluation summaries.

[63] Royal Commission and Board of Inquiry into the Protection and Detention of Children in the Northern Territory (2017). Findings and recommendations.

[64] He VY, Su J-Y, Guthridge S, Malvaso C, Howard D, Williams T, Leach A Hearing and justice: the link between hearing impairment in early childhood and youth offending in Aboriginal children living in remote communities of the Northern Territory, Australia Health Justice 2019;7(1):16, DOI: 10.1186/s40352-019-0097-6. 10.1186/s40352-019-0097-6 PMC6822356 31667630

