# Supplementary material for: Opportunities for prevention: a data-linkage study to inform a public health response to youth offending in the Northern Territory, Australia
Source: BMC Public Health. 2021 Aug 30;21:1600. doi: 10.1186/s12889-021-11645-4 (PMC8404303; doi:10.1186/s12889-021-11645-4)
Supplement: Supplementary file 1 — Additional file 1. [file 12889_2021_11645_MOESM1_ESM.docx]

**Additional file 1**

Table S1: Characteristics of study cohort that have first alleged offence (n=1022), number (and proportion).

|  | Boys | Girls |
| --- | --- | --- |
| Total | 764(100%) | 258(100%) |
| Level of CPS contact | | |
| No contact | 337(44.1%) | 91(35.3%) |
| Notification only | 196(25.7%) | 77(29.8%) |
| Substantiation only | 132(17.3%) | 51(19.8%) |
| Out-of-home care | 99(13.0%) | 39(15.1%) |
| Timing of CPS contact | | |
| N* (one or more unsubstantiated notifications but no substantiations at age 0-4, only) | 31(4.1%) | 10(3.9%) |
| S* (one or more substantiated notifications at age 0-4, only) | 31(4.1%) | 9(3.5%) |
| *N (one or more unsubstantiated notifications but no substantiations at age 5-9, only) | 133(17.4%) | 47(18.2%) |
| *S (one or more substantiated notifications at age 5-9, only) | 63(8.2%) | 23(8.9%) |
| NN (one or more unsubstantiated notifications but no substantiations, at both age 0-4 and age 5-9) | 37(4.8%) | 21(8.1%) |
| SN (one or more substantiated notifications at age 0-4 and unsubstantiated notifications at age 5-9) | 34(4.5%) | 14(5.4%) |
| NS (one or more unsubstantiated notifications at age 0-4 and substantiated notifications at age 5-9) | 46(6.0%) | 23(8.9%) |
| SS (one or more substantiated notifications at both age 0-4 and age 5-9) | 52(6.8%) | 20(7.8%) |
| Indigenous status of mother | | |
| Non-Aboriginal | 60(7.9%) | 23(8.9%) |
| Aboriginal | 704(92.1%) | 235(91.1%) |
| Health district of mother’s residence prior to birth | | |
| Darwin Rural | 96(12.6%) | 17(6.6%) |
| Darwin Urban | 114(14.9%) | 55(21.3%) |
| Katherine | 158(20.7%) | 53(20.5%) |
| East Arnhem | 120(15.7%) | 19(7.4%) |
| Barkly | 79(10.3%) | 23(8.9%) |
| Alice Springs Urban | 51(6.7%) | 30(11.6%) |
| Alice Springs Rural | 146(19.1%) | 61(23.6%) |
| Maternal age at birth | | |
| >=35 | 30(3.9%) | 14(5.4%) |
| <20 | 244(31.9%) | 84(32.6%) |
| 20-24 | 234(30.6%) | 85(32.9%) |
| 25-29 | 167(21.9%) | 52(20.2%) |
| 30-34 | 89(11.6%) | 23(8.9%) |
| Parity | | |
| 0 | 233(30.5%) | 78(30.2%) |
| 1-2 | 328(42.9%) | 123(47.7%) |
| >=3 | 203(26.6%) | 57(22.1%) |
| Low birth weight (< 2500grams) | | |
| No | 660(86.4%) | 223(86.4%) |
| Yes | 104(13.6%) | 35(13.6%) |
| Gestation (in weeks) | | |
| >=37wk | 661(86.5%) | 227(88.0%) |
| <32wk | 21(2.7%) | 3(1.2%) |
| 33-36wk | 82(10.7%) | 28(10.9%) |
| Complication in pregnancy | | |
| No | 706(92.4%) | 237(91.9%) |
| Yes | 58(7.6%) | 21(8.1%) |
| Complication in labour | | |
| No | 489(64.0%) | 177(68.6%) |
| Yes | 275(36.0%) | 81(31.4%) |
| Other obstetric complication | | |
| No | 644(84.3%) | 197(76.4%) |
| Yes | 120(15.7%) | 61(23.6%) |

Table S2: Cumulative incidence (95% confidence intervals) of first alleged offence, by sex, level and timing of CPS contact

|  | Sex | Level and timing | Age 11 | Age 12 | Age 13 | Age 14 | Age 15 | Age 16 | Age 17 | Age 18 |
| --- | --- | --- | --- | --- | --- | --- | --- | --- | --- | --- |
| Level of CPS contact | Boys | No contact with CPS | 0.1(0.0-0.3) | 0.5(0.3-0.9) | 1.7(1.3-2.3) | 4.6(3.9-5.6) | 8.6(7.5-9.9) | 13.8(12.2-15.5) | 18.6(16.7-20.7) | 23.4(21.0-26.1) |
|  |  | Notification only | 0.8(0.4-1.4) | 2.1(1.4-3.1) | 5.9(4.6-7.4) | 11.6(9.7-13.9) | 16.1(13.7-18.8) | 23.4(20.2-26.9) | 29.0(25.0-33.3) | 34.2(29.2-39.8) |
|  |  | Substantiation only | 1.3(0.7-2.3) | 3.6(2.4-5.2) | 7.3(5.5-9.6) | 13.0(10.4-16.2) | 19.0(15.7-23.0) | 27.9(23.5-32.9) | 32.9(27.8-38.6) | 39.5(32.5-47.4) |
|  |  | Out-of-home care | 3.3(1.9-5.7) | 7.4(5.2-10.6) | 16.6(13.0-21.1) | 21.5(17.3-26.6) | 27.6(22.6-33.5) | 35.1(29.1-42.0) | 43.0(35.5-51.3) | 45.5(37.0-54.9) |
|  | Girls | No contact with CPS | 0.04(0.01-0.26) | 0.1(0.0-0.3) | 0.2(0.1-0.5) | 1.1(0.7-1.6) | 2.3(1.7-3.0) | 3.6(2.8-4.6) | 5.1(4.1-6.4) | 6.6(5.3-8.2) |
|  |  | Notification only | 0.1(0.0-0.6) | 0.4(0.2-1.1) | 2.0(1.3-3.2) | 4.4(3.2-6.1) | 8.0(6.2-10.4) | 10.9(8.6-13.8) | 13.0(10.2-16.3) | 15.1(11.7-19.4) |
|  |  | Substantiation only | 0 | 1.1(0.5-2.3) | 3.0(1.9-4.9) | 6.5(4.6-9.1) | 10.1(7.5-13.5) | 12.7(9.6-16.7) | 13.4(10.1-17.7) | 15.3(10.8-21.4) |
|  |  | Out-of-home care | 0.8(0.2-2.3) | 3.6(2.1-6.1) | 5.2(3.3-8.2) | 8.5(5.8-12.4) | 12.7(9.1-17.6) | 15.3(11.0-21.0) | 15.3(11.0-21.0) | 18.6(13.0-26.2) |
| Timing of CPS contact | Boys | 1.N* | 0.4(0.1-2.8) | 0.8(0.2-3.3) | 3.3(1.6-6.9) | 6.8(4.0-11.5) | 9.8(6.1-15.4) | 17.2(11.7-24.8) | 24.2(17.0-33.8) | 26.3(18.4-36.6) |
|  |  | 2.S* | 0.5(0.1-3.7) | 2.2(0.8-5.7) | 4.8(2.4-9.3) | 7.1(4.0-12.6) | 10.7(6.5-17.4) | 20.9(14.2-30.0) | 33.2(23.6-45.3) | 33.2(23.6-45.3) |
|  |  | 3.*N | 0.7(0.3-1.6) | 2.0(1.2-3.3) | 6.0(4.4-8.0) | 11.8(9.4-14.7) | 17.5(14.4-21.2) | 25.7(21.6-30.3) | 30.6(25.7-36.2) | 37.8(31.2-45.4) |
|  |  | 4.*S | 1.4(0.6-3.3) | 2.6(1.3-4.9) | 6.3(4.1-9.6) | 10.3(7.3-14.5) | 17.8(13.4-23.5) | 25.6(19.9-32.5) | 30.7(24.0-38.6) | 37.5(28.4-48.3) |
|  |  | 5.NN | 1.1(0.4-3.4) | 3.3(1.6-6.5) | 8.5(5.4-13.3) | 16.1(11.4-22.6) | 18.9(13.6-26.1) | 23.1(16.6-31.7) | 29.4(20.1-41.8) | 29.4(20.1-41.8) |
|  |  | 6.SN | 1.5(0.5-4.5) | 3.8(1.8-7.8) | 7.5(4.3-12.9) | 17.3(11.6-25.5) | 25.1(17.6-35.0) | 30.2(21.6-41.3) | 32.4(23.2-44.0) | 43.7(29.0-61.8) |
|  |  | 7.NS | 4.5(2.4-8.6) | 8.0(4.9-13.0) | 20.8(15.0-28.4) | 29.7(22.3-38.8) | 31.0(23.4-40.4) | 37.7(28.2-49.2) | 46.0(33.2-61.0) | 46.0(33.2-61.0) |
|  |  | 8.SS | 2.4(1.0-5.7) | 10.5(6.8-16.1) | 18.2(13.0-25.1) | 24.5(18.2-32.5) | 32.4(24.5-42.1) | 45.7(35.2-57.7) | 48.3(37.2-60.7) | 58.6(39.6-78.7) |
|  | Girls | 1.N* | 0 | 0 | 0 | 1.3(0.3-4.9) | 3.8(1.6-9.0) | 7.0(3.5-13.8) | 9.8(5.2-17.8) | 9.8(5.2-17.8) |
|  |  | 2.S* | 0 | 0 | 0.6(0.1-4.3) | 1.4(0.3-5.6) | 5.7(2.7-11.6) | 5.7(2.7-11.6) | 7.7(3.7-15.9) | 13.5(5.4-31.8) |
|  |  | 3.*N | 0 | 0.1(0.0-1.1) | 1.9(1.0-3.3) | 4.7(3.2-7.0) | 8.3(6.0-11.3) | 10.4(7.8-14.0) | 10.9(8.1-14.6) | 14.0(10.0-19.4) |
|  |  | 4.*S | 0 | 1.0(0.3-3.2) | 2.9(1.5-5.8) | 5.2(3.1-8.9) | 9.6(6.3-14.4) | 11.1(7.4-16.4) | 11.1(7.4-16.4) | 11.1(7.4-16.4) |
|  |  | 5.NN | 0.4(0.1-2.8) | 1.8(0.7-4.6) | 4.4(2.2-8.7) | 6.1(3.2-11.3) | 10.2(6.0-17.0) | 14.9(9.1-24.1) | 21.6(13.4-33.7) | 26.2(15.7-41.7) |
|  |  | 6.SN | 0.9(0.2-3.7) | 2.5(1.1-5.9) | 4.7(2.3-9.2) | 7.5(4.1-13.6) | 7.5(4.1-13.6) | 11.9(6.4-21.5) | 11.9(6.4-21.5) | 16.1(8.2-30.2) |
|  |  | 7.NS | 0 | 3.2(1.2-8.3) | 6.0(2.9-12.2) | 16.1(9.9-25.7) | 26.3(17.6-38.2) | 30.9(21.1-43.9) | 30.9(21.1-43.9) | 30.9(21.1-43.9) |
|  |  | 8.SS | 0.5(0.1-3.7) | 4.8(2.4-9.4) | 7.2(4.0-12.8) | 12.9(7.9-20.8) | 14.7(9.0-23.4) | 23.0(14.0-36.3) | 23.0(14.0-36.3) | 23.0(14.0-36.3) |

Note: ****** (no record of CPS contact before age 10); **N*** (one or more unsubstantiated notifications but no substantiations at age 0-4, only) ; **S*** (one or more substantiated notifications at age 0-4, only) ; ***N** (one or more unsubstantiated notifications but no substantiations at age 5-9, only); ***S** (one or more substantiated notifications at age 5-9, only); **NN** (one or more unsubstantiated notifications but no substantiations, at both age 0-4 and age 5-9) ; **SN** (one or more substantiated notifications at age 0-4 and unsubstantiated notifications at age 5-9) ; **NS** (one or more unsubstantiated notifications at age 0-4 and substantiated notifications at age 5-9) ; **SS** (one or more substantiated notifications at both age 0-4 and age 5-9).

Table S3: Cumulative incidence (95% confidence intervals) of first alleged offence, by sex, prenatal and perinatal characteristics

|  | Boys | Girls |
| --- | --- | --- |
| All children | 28.9%(26.8-31.1) | 10.0%(8.7-11.4) |
| Indigenous status of mother | | |
| Non-Aboriginal | 19.3%(14.5-25.3) | 8.0%(5.1-12.2) |
| Aboriginal | 30.2%(27.9-32.6) | 10.2%(8.9-11.7) |
| Health district of mother’s residence prior to birth | | |
| Darwin Rural | 24.7%(19.8-30.7) | 3.8%(2.2-6.6) |
| Darwin Urban | 22.9%(18.9-27.7) | 11.7%(8.7-15.7) |
| Katherine | 31.7%(27.0-36.9) | 11.0%(8.3-14.5) |
| East Arnhem | 29.9%(24.6-36.1) | 4.7%(2.8-7.8) |
| Barkly | 41.6%(33.8-50.4) | 14.5%(9.5-21.7) |
| Alice Springs Urban | 20.8%(15.8-27.1) | 12.9%(8.7-18.8) |
| Alice Springs Rural | 35.9%(30.3-42.1) | 16.8%(12.8-21.8) |
| Maternal age at birth | | |
| >=35 | 26.2%(17.7-37.7) | 8.4%(4.9-14.3) |
| <20 | 31.1%(27.2-35.3) | 10.9%(8.5-13.8) |
| 20-24 | 27.5%(24.0-31.4) | 11.8%(9.3-14.9) |
| 25-29 | 28.5%(24.3-33.1) | 9.0%(6.7-12.0) |
| 30-34 | 28.9%(22.7-36.3) | 5.7%(3.7-8.5) |
| Parity | | |
| 0 | 27.1%(23.7-30.9) | 8.7%(6.8-11.1) |
| 1-2 | 29.1%(25.9-32.7) | 12.4%(10.2-15.0) |
| >=3 | 31.2%(27.1-35.8) | 7.7%(5.8-10.1) |
| Low birth weight (< 2500 grams) | | |
| No | 28.6%(26.3-31.0) | 9.8%(8.5-11.3) |
| Yes | 31.5%(26.0-37.7) | 11.1%(7.7-15.8) |
| Gestation (in weeks) | | |
| >=37wk | 28.8%(26.5-31.2) | 10.1%(8.8-11.7) |
| <32wk | 30.4%(19.6-45.3) | 4.8%(1.5-14.8) |
| 33-36wk | 30.1%(24.3-36.9) | 9.8%(6.5-14.5) |
| Complication in pregnancy | | |
| No | 29.0%(26.9-31.3) | 9.9%(8.6-11.4) |
| Yes | 27.4%(20.5-35.9) | 10.2%(6.1-17.1) |
| Complication in labour | | |
| No | 30.5%(27.8-33.3) | 10.3%(8.8-12.1) |
| Yes | 26.2%(23.0-29.7) | 9.5%(7.4-12.2) |
| Other obstetric complication | | |
| No | 29.0%(26.7-31.4) | 9.2%(7.9-10.6) |
| Yes | 28.6%(23.5-34.5) | 13.9%(10.4-18.5) |

Table S4: Univariable and multivariable hazard ratios at different ages by sex, level and timing of CPS contact

|  | Sex |  | Level and timing | Age 11 | Age 12 | Age 13 | Age 14 | Age 15 | Age 16 | Age 17 |
| --- | --- | --- | --- | --- | --- | --- | --- | --- | --- | --- |
| Level of CPS contact | Boys | Univariate | Notification only | 4.8(3.0-7.8) | 3.0(2.3-4.1) | 2.3(1.9-2.9) | 1.9(1.6-2.3) | 1.6(1.3-1.9) | 1.3(1.0-1.7) | 1.1(0.8-1.5) |
|  |  |  | Substantiation only | 6.7(4.1-11.0) | 3.9(2.9-5.3) | 2.9(2.3-3.6) | 2.2(1.8-2.8) | 1.8(1.4-2.3) | 1.4(1.1-1.9) | 1.2(0.9-1.7) |
|  |  |  | Out-of-home care | 14.0(8.5-23.0) | 6.7(4.9-9.2) | 4.3(3.4-5.6) | 3.1(2.4-4.0) | 2.2(1.7-2.9) | 1.6(1.1-2.3) | 1.2(0.8-1.9) |
|  |  | Multivariable | Notification only | 5.0(3.1-8.0) | 3.1(2.3-4.2) | 2.3(1.9-2.9) | 1.9(1.6-2.3) | 1.6(1.3-1.9) | 1.3(1.0-1.7) | 1.1(0.8-1.5) |
|  |  |  | Substantiation only | 6.9(4.2-11.2) | 3.9(2.9-5.4) | 2.9(2.3-3.6) | 2.2(1.8-2.8) | 1.8(1.4-2.2) | 1.4(1.0-1.9) | 1.2(0.8-1.7) |
|  |  |  | Out-of-home care | 13.7(8.3-22.5) | 6.6(4.8-9.0) | 4.2(3.3-5.5) | 3.0(2.4-3.9) | 2.2(1.6-2.9) | 1.5(1.1-2.2) | 1.2(0.8-1.9) |
|  | Girls | Univariate | Notification only | 8.0(3.0-21.6) | 5.1(2.8-9.5) | 4.0(2.6-6.0) | 3.3(2.4-4.5) | 2.6(1.9-3.7) | 2.1(1.4-3.3) | 1.8(1.0-3.2) |
|  |  |  | Substantiation only | 17.4(6.3-47.6) | 8.2(4.4-15.3) | 5.3(3.4-8.2) | 3.8(2.6-5.5) | 2.6(1.7-3.9) | 1.7(0.9-3.1) | 1.2(0.5-2.8) |
|  |  |  | Out-of-home care | 35.7(13.4-95.0) | 13.5(7.3-24.9) | 7.6(4.8-12.1) | 4.9(3.2-7.4) | 2.8(1.8-4.5) | 1.5(0.7-3.0) | 0.8(0.3-2.4) |
|  |  | Multivariable | Notification only | 6.8(2.5-18.2) | 4.4(2.4-8.1) | 3.4(2.2-5.3) | 2.8(2.0-4.0) | 2.3(1.7-3.2) | 1.9(1.2-2.9) | 1.6(0.9-2.8) |
|  |  |  | Substantiation only | 16.3(6.0-44.0) | 7.8(4.2-14.5) | 5.1(3.2-7.9) | 3.7(2.5-5.3) | 2.5(1.7-3.7) | 1.6(0.9-3.0) | 1.1(0.5-2.7) |
|  |  |  | Out-of-home care | 27.6(10.5-72.8) | 10.7(5.8-19.9) | 6.2(3.9-10.0) | 4.0(2.6-6.2) | 2.4(1.5-3.8) | 1.2(0.6-2.4) | 0.6(0.2-2.0) |
| Timing of CPS contact | Boys | Univariate | 1.N* | 1.7(0.6-5.2) | 1.5(0.8-2.9) | 1.4(0.9-2.2) | 1.3(0.9-1.9) | 1.2(0.8-1.8) | 1.2(0.7-1.9) | 1.1(0.6-2.0) |
|  |  |  | 2.S* | 2.8(1.0-7.8) | 2.2(1.2-4.1) | 1.9(1.2-3.0) | 1.8(1.2-2.5) | 1.6(1.1-2.4) | 1.5(0.9-2.4) | 1.4(0.8-2.5) |
|  |  |  | 3.*N | 4.7(2.8-8.1) | 3.2(2.3-4.4) | 2.5(2.0-3.1) | 2.1(1.7-2.5) | 1.7(1.4-2.2) | 1.5(1.1-2.0) | 1.3(0.9-1.9) |
|  |  |  | 4.*S | 6.1(3.3-11.4) | 3.5(2.4-5.2) | 2.5(1.9-3.4) | 2.0(1.5-2.6) | 1.6(1.1-2.1) | 1.3(0.8-1.9) | 1.0(0.7-1.7) |
|  |  |  | 5.NN | 8.9(4.9-16.5) | 4.1(2.7-6.3) | 2.6(1.8-3.8) | 1.9(1.3-2.7) | 1.3(0.8-2.0) | 0.9(0.5-1.6) | 0.7(0.4-1.4) |
|  |  |  | 6.SN | 8.5(4.2-17.2) | 4.7(3.0-7.4) | 3.3(2.3-4.8) | 2.5(1.7-3.6) | 1.9(1.2-3.0) | 1.5(0.9-2.6) | 1.2(0.6-2.4) |
|  |  |  | 7.NS | 19.1(11.0-33.2) | 8.3(5.7-12.2) | 5.1(3.6-7.1) | 3.5(2.4-5.0) | 2.3(1.5-3.6) | 1.6(0.9-2.8) | 1.2(0.6-2.3) |
|  |  |  | 8.SS | 17.0(9.6-30.0) | 8.5(5.9-12.4) | 5.7(4.1-7.8) | 4.2(3.0-5.8) | 3.1(2.1-4.5) | 2.3(1.4-3.8) | 1.8(1.0-3.3) |
|  |  | Multivariable | 1.N* | 1.7(0.6-5.1) | 1.4(0.7-2.8) | 1.3(0.8-2.1) | 1.2(0.8-1.8) | 1.2(0.8-1.7) | 1.1(0.7-1.8) | 1.0(0.6-1.9) |
|  |  |  | 2.S* | 2.7(0.9-7.6) | 2.2(1.1-4.1) | 1.9(1.2-3.0) | 1.7(1.2-2.5) | 1.6(1.1-2.4) | 1.5(0.9-2.4) | 1.4(0.8-2.5) |
|  |  |  | 3.*N | 4.9(2.9-8.4) | 3.3(2.3-4.5) | 2.6(2.0-3.2) | 2.1(1.7-2.6) | 1.8(1.4-2.3) | 1.5(1.1-2.0) | 1.3(0.9-1.9) |
|  |  |  | 4.*S | 6.0(3.2-11.1) | 3.4(2.3-5.1) | 2.5(1.8-3.3) | 1.9(1.5-2.6) | 1.5(1.1-2.1) | 1.2(0.8-1.8) | 1.0(0.6-1.6) |
|  |  |  | 5.NN | 10.2(5.5-18.7) | 4.6(3.0-6.9) | 2.8(2.0-4.1) | 2.0(1.4-2.9) | 1.4(0.9-2.1) | 0.9(0.5-1.7) | 0.7(0.3-1.4) |
|  |  |  | 6.SN | 9.4(4.7-18.8) | 5.0(3.1-7.8) | 3.4(2.3-5.0) | 2.6(1.8-3.7) | 1.9(1.2-3.0) | 1.5(0.8-2.5) | 1.2(0.6-2.3) |
|  |  |  | 7.NS | 20.9(12.0-36.5) | 8.8(6.0-13.0) | 5.3(3.8-7.5) | 3.6(2.5-5.2) | 2.4(1.5-3.7) | 1.5(0.9-2.7) | 1.1(0.5-2.3) |
|  |  |  | 8.SS | 18.5(10.5-32.7) | 9.0(6.2-13.1) | 5.9(4.3-8.2) | 4.3(3.1-6.0) | 3.1(2.1-4.6) | 2.2(1.3-3.7) | 1.7(0.9-3.2) |
|  | Girls | Univariate | 1.N* | 1.1(0.1-15.8) | 1.4(0.3-6.8) | 1.5(0.5-4.4) | 1.6(0.8-3.5) | 1.8(0.9-3.4) | 1.9(0.8-4.4) | 2.0(0.7-5.9) |
|  |  |  | 2.S* | 2.5(0.2-27.4) | 2.2(0.5-9.4) | 2.0(0.8-5.4) | 1.9(0.9-4.0) | 1.8(0.9-3.7) | 1.7(0.7-4.5) | 1.7(0.5-5.8) |
|  |  |  | 3.*N | 8.2(2.7-25.1) | 5.0(2.5-10.0) | 3.8(2.4-6.1) | 3.1(2.1-4.5) | 2.4(1.7-3.6) | 1.9(1.1-3.3) | 1.6(0.8-3.2) |
|  |  |  | 4.*S | 22.4(6.6-76.0) | 8.6(4.0-18.3) | 4.9(2.8-8.5) | 3.2(2.0-5.2) | 1.9(1.0-3.4) | 1.0(0.3-3.0) | 0.5(0.1-4.0) |
|  |  |  | 5.NN | 15.1(4.2-55.0) | 9.2(4.1-20.5) | 6.9(3.9-12.3) | 5.5(3.4-9.0) | 4.3(2.5-7.4) | 3.4(1.6-7.0) | 2.7(1.1-7.1) |
|  |  |  | 6.SN | 27.3(8.8-84.9) | 10.5(4.9-22.6) | 6.0(3.2-11.3) | 3.9(2.2-7.2) | 2.3(1.2-4.6) | 1.2(0.5-3.2) | 0.6(0.1-3.0) |
|  |  |  | 7.NS | 44.2(12.5-155.4) | 21.3(10.0-45.4) | 13.9(8.1-23.9) | 10.0(6.2-16.3) | 6.9(3.8-12.7) | 4.5(1.7-11.8) | 3.1(0.8-12.5) |
|  |  |  | 8.SS | 58.9(19.7-176.6) | 20.1(10.0-40.7) | 10.8(6.1-18.9) | 6.6(3.8-11.4) | 3.5(1.8-7.0) | 1.5(0.4-5.4) | 0.6(0.0-11.2) |
|  |  | Multivariable | 1.N* | 1.2(0.1-15.0) | 1.3(0.3-6.3) | 1.4(0.5-4.0) | 1.5(0.7-3.1) | 1.5(0.8-3.0) | 1.6(0.7-3.7) | 1.7(0.6-5.0) |
|  |  |  | 2.S* | 2.5(0.2-26.5) | 2.2(0.5-9.3) | 2.0(0.8-5.4) | 1.9(0.9-4.0) | 1.8(0.9-3.7) | 1.7(0.6-4.4) | 1.6(0.4-5.6) |
|  |  |  | 3.*N | 6.9(2.3-20.8) | 4.3(2.2-8.5) | 3.2(2.0-5.2) | 2.6(1.8-3.8) | 2.1(1.4-3.0) | 1.6(0.9-2.8) | 1.3(0.6-2.7) |
|  |  |  | 4.*S | 21.1(6.3-70.0) | 8.2(3.8-17.4) | 4.7(2.7-8.2) | 3.1(1.9-5.0) | 1.8(1.0-3.2) | 0.9(0.3-2.9) | 0.4(0.0-5.2) |
|  |  |  | 5.NN | 13.7(3.8-48.9) | 8.4(3.8-18.9) | 6.4(3.5-11.5) | 5.2(3.1-8.6) | 4.1(2.4-7.0) | 3.1(1.5-6.6) | 2.5(1.0-6.7) |
|  |  |  | 6.SN | 20.5(6.7-62.6) | 8.2(3.8-17.6) | 4.8(2.5-9.0) | 3.2(1.7-5.8) | 1.9(1.0-3.7) | 1.0(0.4-2.5) | 0.5(0.1-2.3) |
|  |  |  | 7.NS | 40.0(11.5-139.7) | 19.9(9.3-42.7) | 13.3(7.6-23.2) | 9.7(5.9-16.1) | 6.7(3.6-12.6) | 4.4(1.6-11.8) | 3.0(0.7-12.9) |
|  |  |  | 8.SS | 54.1(18.1-162.0) | 19.6(9.6-40.0) | 10.9(6.1-19.4) | 6.9(3.9-12.0) | 3.7(1.9-7.6) | 1.6(0.4-6.0) | 0.6(0.0-14.5) |

Note: ****** (no record of CPS contact before age 10); **N*** (one or more unsubstantiated notifications but no substantiations at age 0-4, only) ; **S*** (one or more substantiated notifications at age 0-4, only) ; ***N** (one or more unsubstantiated notifications but no substantiations at age 5-9, only); ***S** (one or more substantiated notifications at age 5-9, only); **NN** (one or more unsubstantiated notifications but no substantiations, at both age 0-4 and age 5-9) ; **SN** (one or more substantiated notifications at age 0-4 and unsubstantiated notifications at age 5-9) ; **NS** (one or more unsubstantiated notifications at age 0-4 and substantiated notifications at age 5-9) ; **SS** (one or more substantiated notifications at both age 0-4 and age 5-9)

Table S5: Multivariable flexible parametric survival model adjusted for ‘cohort fixed-effects’

|  | Model 1 (level of CPS contact) | | Model 2 (timing of CPS contact) | |
| --- | --- | --- | --- | --- |
|  | Boys | Girls | Boys | Girls |
| Royston R^2^ | 0.588(0.551-0.621) | 0.673(0.619-0.718) | 0.577(0.539-0.613) | 0.688(0.615-0.712) |
|  | Hazard ratio (95% CI) | | | |
| Indigenous status of mother | | | | |
| Non-Aboriginal | 1.0 (reference group) | | | |
| Aboriginal | 1.3(1.0-1.8)* | 1.2(0.7-1.9) | 1.3(1.0-1.8)* | 1.2(0.7-1.9) |
| Health district of mother’s residence prior to birth | | | | |
| Darwin Rural | 1.0 (reference group) | | | |
| Darwin Urban | 1.3(1.0-1.7)# | 3.6(2.0-6.3)*** | 1.3(1.0-1.7) | 3.3(1.9-5.8)*** |
| Katherine | 1.7(1.3-2.1)*** | 2.5(1.4-4.5)** | 1.7(1.3-2.1)*** | 2.5(1.4-4.5)** |
| East Arnhem | 1.5(1.1-1.9)** | 1.2(0.6-2.3) | 1.5(1.1-1.9)** | 1.2(0.6-2.4) |
| Barkly | 2.4(1.8-3.2)*** | 3.2(1.7-6.0)*** | 2.4(1.7-3.2)*** | 3.0(1.6-5.8)*** |
| Alice Springs Urban | 1.3(0.9-1.8) | 3.9(2.1-7.2)*** | 1.2(0.9-1.7) | 3.9(2.1-7.3)*** |
| Alice Springs Rural | 2.0(1.5-2.6)*** | 3.8(2.2-6.7)*** | 2.0(1.6-2.7)*** | 4.1(2.3-7.1)*** |
| Maternal age at birth | | | | |
| >=35 | 1.0 (reference group) | | | |
| <20 | 1.6(1.1-2.5)* | 1.4(0.7-2.6) | 1.6(1.1-2.5)* | 1.3(0.7-2.5) |
| 20-24 | 1.5(1.0-2.2)# | 1.2(0.7-2.2) | 1.5(1.0-2.2)# | 1.2(0.6-2.1) |
| 25-29 | 1.2(0.8-1.8) | 0.9(0.5-1.7) | 1.3(0.8-1.9) | 0.9(0.5-1.6) |
| 30-34 | 1.4(0.9-2.1) | 0.8(0.4-1.5) | 1.4(0.9-2.1) | 0.7(0.4-1.5) |
| Parity | | | | |
| '0 | 1.0 (reference group) | | | |
| '1-2 | 1.2(1.0-1.4) | 1.6(1.1-2.2)** | 1.1(0.9-1.4) | 1.7(1.2-2.3)** |
| >=3 | 1.4(1.1-1.8)** | 1.4(0.9-2.2) | 1.4(1.1-1.8)* | 1.4(0.9-2.2) |
| LBW? |  |  |  |  |
| No |  |  |  |  |
| Yes | 1.2(0.9-1.6) | 1.2(0.8-1.9) | 1.2(0.9-1.6) | 1.3(0.8-2.0) |
| Gestation (in weeks) |  |  |  |  |
| >=37wk | 1.0 (reference group) | | | |
| <32wk | 0.8(0.5-1.3) | 0.4(0.1-1.2)# | 0.8(0.5-1.3) | 0.3(0.1-1.1)# |
| 33-36wk | 0.8(0.6-1.1) | 0.8(0.5-1.3) | 0.8(0.6-1.1) | 0.8(0.5-1.2) |
| Complication in pregnancy | | | | |
| No | 1.0 (reference group) | | | |
| Yes | 1.0(0.8-1.3) | 1.2(0.8-1.9) | 1.0(0.8-1.4) | 1.3(0.8-2.0) |
| Complication in labour | | | | |
| No | 1.0 (reference group) | | | |
| Yes | 0.9(0.8-1.1) | 0.8(0.6-1.1) | 0.9(0.8-1.1) | 0.8(0.6-1.1) |
| Other obstetric complication | | | | |
| No | 1.0 (reference group) | | | |
| Yes | 1.0(0.8-1.2) | 1.5(1.1-2.1)** | 1.0(0.8-1.2) | 1.5(1.1-2.1)** |
|  | Baseline hazard (log hazard scale) Coefficient (95% CI) | | | |
| Level of CPS contact | | | | |
| Notification only | 0.9(0.7-1.2)*** | 1.2(0.8-1.6)*** |  |  |
| Substantiation only | 1.2(0.9-1.4)*** | 1.6(1.2-2.0)*** |  |  |
| Out-of-home care | 1.6(1.4-1.9)*** | 1.8(1.4-2.3)*** |  |  |
| Restricted cubic splines of attained age | | | | |
| _rcs1 | 1.5(1.3-1.6)*** | 1.7(1.4-2.0)*** |  |  |
| _rcs2 | -0.0(-0.1-0.0) | 0.1(0.0-0.2)** |  |  |
| _rcs3 | 0.1(0.0-0.1)*** | 0.1(0.1-0.2)*** |  |  |
| Interactions between attained age and level of CPS contact | | | | |
| _rcs_notifications | -0.4(-0.6- -0.2)*** | -0.3(-0.7- -0.0)* |  |  |
| _rcs_substantiations | -0.5(-0.7- -0.3)*** | -0.6(-1.0- -0.3)*** |  |  |
| _rcs_OHC | -0.7(-0.9- -0.5)*** | -0.8(-1.1- -0.5)*** |  |  |
| _cons | -4.0(-4.6- -3.5)*** | -5.7(-6.7- -4.8)*** |  |  |
| Timing of CPS contact | | | | |
| 1.N* |  |  | 0.3(-0.2-0.8) | 0.3(-0.6-1.3) |
| 2.S* |  |  | 0.7(0.2-1.2)** | 0.7(-0.2-1.6) |
| 3.*N |  |  | 1.0(0.7-1.3)*** | 1.1(0.7-1.6)*** |
| 4.*S |  |  | 1.0(0.7-1.3)*** | 1.5(1.0-2.1)*** |
| 5.NN |  |  | 1.3(0.9-1.6)*** | 1.8(1.2-2.4)*** |
| 6.SN |  |  | 1.4(1.0-1.8)*** | 1.5(0.9-2.2)*** |
| 7.NS |  |  | 1.9(1.6-2.3)*** | 2.5(2.0-3.1)*** |
| 8.SS |  |  | 2.0(1.6-2.3)*** | 2.4(1.8-2.9)*** |
| Restricted cubic splines of attained age | | | | |
| _rcs1 |  |  | 1.5(1.4-1.7)*** | 1.7(1.4-2.0)*** |
| _rcs2 |  |  | -0.0(-0.1-0.0) | 0.1(0.0-0.2)** |
| _rcs3 |  |  | 0.1(0.0-0.1)*** | 0.1(0.1-0.2)*** |
| Interactions between attained age and timing of CPS contact | | | | |
| _rcs_N* |  |  | -0.1(-0.6-0.3) | 0.1(-0.7-0.9) |
| _rcs_S* |  |  | -0.2(-0.6-0.2) | -0.1(-0.9-0.7) |
| _rcs_*N |  |  | -0.4(-0.6--0.1)*** | -0.4(-0.8--0.0)* |
| _rcs_*S |  |  | -0.5(-0.7--0.2)*** | -0.8(-1.2--0.3)*** |
| _rcs_NN |  |  | -0.7(-1.0--0.5)*** | -0.4(-0.8-0.1)# |
| _rcs_SN |  |  | -0.6(-0.9--0.3)*** | -0.8(-1.1--0.4)*** |
| _rcs_NS |  |  | -0.8(-1.0--0.6)*** | -0.6(-1.1--0.1)* |
| _rcs_SS |  |  | -0.6(-0.9--0.4)*** | -0.9(-1.2--0.5)*** |
| _cons |  |  | -4.0(-4.6--3.5)*** | -5.7(-6.7--4.8)*** |

Note: ****** (no record of CPS contact before age 10); **N*** (one or more unsubstantiated notifications but no substantiations at age 0-4, only) ; **S*** (one or more substantiated notifications at age 0-4, only) ; ***N** (one or more unsubstantiated notifications but no substantiations at age 5-9, only); ***S** (one or more substantiated notifications at age 5-9, only); **NN** (one or more unsubstantiated notifications but no substantiations, at both age 0-4 and age 5-9) ; **SN** (one or more substantiated notifications at age 0-4 and unsubstantiated notifications at age 5-9) ; **NS** (one or more unsubstantiated notifications at age 0-4 and substantiated notifications at age 5-9) ; **SS** (one or more substantiated notifications at both age 0-4 and age 5-9).
